# Neuropsychiatric Disturbance in Huntington’s Disease: Approach to Management

**DOI:** 10.1192/j.eurpsy.2022.1208

**Published:** 2022-09-01

**Authors:** J. Jay, V. Kumar, P. Bidkhanian, E. Garrels, Y. Segal, B. Susaimanickam

**Affiliations:** BronxCare Health System, Psychiatry, Bronx, United States of America

**Keywords:** Neuropsychiatry, Huntington’s disease, Psychosis

## Abstract

**Introduction:**

Huntington’s Disease (HD) is an autosomal dominant, neurodegenerative condition with a prevalence of 10.6-13.7 per 100,000, caused by the trinucleotide CAG (cytosine, adenine, guanine) repeat expansion in the HTT gene. HD is characterized by a range of motor, cognitive, and psychiatric symptoms, the latter of which usually manifest prior to the onset of motor or cognitive disturbances. Amongst psychiatric symptoms, changes in personality are most common, followed by depression. Psychosis has a higher prevalence in those with early-onset HD.

**Objectives:**

This case report aims to demonstrate an apporach to the management of neuropsychiatric disturbances in HD as well as expose the need for development of an evidence-based apprach to treatment.

**Methods:**

PubMed was searched for the criteria Huntington’s Disease AND Psychosis, with a secondary search for Management of Psychosis in Huntington’s Disease.

**Results:**

The patient is a 54-year-old male with no psychiatric history and reported past medical history of Huntington’s Disease, diagnosed one month ago. He was brought to the Psychiatric ED due to agitation and disorganized behavior at home. On admission, he demonstrated disorganized behavior, grandiose delusions, neurocognitive deficits, and reported auditory hallucinations. With the initiation of tetrabenazine and risperidone his psychiatric symptoms improved and he was able to be discharged to a long-term care facility.

**Conclusions:**

Literature is scarce regarding treatment of psychiatric manifestations of HD. We catered our approach towards safe and effective symptoms management in a multidisciplinary manner. Further research is required to reach an evidence-based consensus as well as develop specific guidelines for managing psychiatric conditions related to HD.

**Disclosure:**

No significant relationships.

